# Sperm tendency to agglutinate in motile bundles in relation to sperm competition and fertility duration in chickens

**DOI:** 10.1038/s41598-022-22049-8

**Published:** 2022-11-07

**Authors:** M. A. M. Sayed, Hanan H. Abd-Elhafeez, O. S. Afifi, M. W. Marzouk, Taymour M. El-Sherry

**Affiliations:** 1 Department of Poultry Production, Faculty of Agriculture, Assiut University, Assiut, 71526 Egypt; 2 Department of Cell and Tissues, Faculty of Veterinary Medicine, Assiut University, Assiut, 71526 Egypt; 3 Department of Poultry Production, Faculty of Agriculture, New Valley University, Kharga, 72712 Egypt; 4 Department of Theriogenology, Faculty of Veterinary Medicine, Assiut University, Assiut, 71526 Egypt

**Keywords:** Biological techniques, Behavioural methods, Imaging, Microscopy, Structure determination

## Abstract

A unique sperm behavior was observed in Egyptian chickens. Sperm showed a tendency to agglutinate forming motile thread-like bundles. Sperm agglutination behavior, kinematics, and some morphometric measures were studied in relation to sperm competition and fertility duration in Sharkasi and Dandarawi chickens. Sperm tendency to agglutinate was assessed by examining sperm morphology using scanning electron microscopy, Acridine orange-stained semen smears using fluorescence microscopy, and recording videos of sperm under phase contrast microscope. Sperm velocity and morphometric measures were evaluated using image-J software. To assess sperm competition, Sharkasi and Dandarawi hens were artificially inseminated by semen pools possessing equal number of Sharaksi and Dandarawi sperm. Artificial insemination was repeated ten times. The eggs obtained were incubated, and the hatchlings were discriminated as descending from Sharkasi or Dandarawi fathers according to their phenotype. To assess the fertility duration, Sharkasi and Dandarawi hens were inseminated by semen collected from roosters of the same strain. Eggs were collected for a period of 28 days post-insemination and incubated. Sharkasi spermatozoa showed higher tendency to agglutinate forming longer and thicker motile bundles. No significant differences were observed in sperm curvilinear and straight line velocity and in sperm morphometric measures between Sharkasi and Dandarawi chickens. Sharkasi roosters fathered 81.6% and 67.7% of the hatchlings produced by Sharkasi and Dandarawi mothers, respectively. The fertility period in Sharkasi and Dandarawi was 22 and 14 days, respectively. We suggest that the differences seen in sperm competitiveness and fertility duration can be attributed to sperm agglutination behavior.

## Introduction

Sharkasi and Dandarawi are among popular Egyptian chicken strains reared in rural areas because they are well adapted to the prevalent environmental conditions and common diseases. Sharkasi chickens have three different phenotypes because of the presence of the naked neck gene. These phenotypes are; (a) homozygous (Na/Na), (b) heterozygous (Na/na) and (c) normally feathered (na/na). Crawford^1^ showed that the naked neck gene (Na), a marker gene, is incompletely dominant. The heterozygous (Nana) can be defined by the feathers tuft on the ventral side of the neck above the crop. However, the homozygous do not have this tuft or just a few small feathers.

It is known that sperm agglutination in humans can cause infertility^2^. This is because the sperm must move freely and not be restricted by a mass that impedes its ability to swim to reach the egg. However, in other mammals such as platypus and echidna, sperm agglutinate parallel to each other forming motile bundles consist of approximately 100 cells which increase their swimming velocity and consequently their ability to compete with sperm from other males^3^. In birds, sperm agglutinate head-to-head and remain in a quiescent state while being stored in the uterovaginal junction^4–6^.

Sperm competition can be defined as the competition between spermatozoa from ejaculates of different males to fertilize the ovum^7,8^ (Wedell, 2010). This competition occurs naturally in birds that mate with more than one male^9^. It is believed that this competition is affected by many factors including; sperm concentration in the ejaculate, the timing of insemination, the interval between consecutive inseminations, sperm quality and its fertilizing capacity.

Several studies have linked between some sperm characteristics and fertilizing ability in domestic fowls, such as sperm concentration, motility, membrane integrity, viability… etc. However, these relationships have been assessed in a non-competitive situation (Birkhead et al.^9^. In turkeys, Donoghue et al.^10^ could not observe any correlation between semen traits and sperm characteristics (semen volume, sperm concentration, sperm viability, membrane integrity, and subjective sperm motility) and paternity. However, other studies have shown that sperm number influence paternity proportions after heterospermic insemination (Martin et al.^11^. Sperm mobility was introduced as a trait that is normally distributed among males and predicts fertility and paternity outcomes^9,10,12^. Donoghue et al.^10^ reported that sperm mobility is composed of several parameters, including sperm motility. The assay of sperm mobility measures the ability of sperm to swim against resistance into a dense medium (Accudenz) at the hen’s body temperature (41.8 °C) and therefore, objectively measures the proportion of sperm with a vigorous progressive motility. Although mobile sperm must be motile, not all motile sperm are mobile^13^. For example, straight-line velocity (VSL) must be > 30 µm/s for sperm to penetrate an Accudenz solution^13^.

Many hypotheses have been put forward to elucidate the mechanism by which sperm competition works. In several bird species, it was observed that a large number of offspring descends from the last male’s insemination, which is known as "last male sperm precedence"^14^. Therefore, some researchers attributed this phenomenon to sperm being stored in the sperm storage tubules (SSTs) in a form of stratifications and that the last layers of stored sperm are the first to emerge from sperm nests for fertilization "last in, first out"^15^, On the contrary, King et al.^16^ demonstrated, by inseminating the hens with fluorescence stained spermatozoa, that sperm from different males are stored in different SSTs and that only a small percentage of the SSTs contained sperm from different males. This means that sperm from different ejaculates are segregated in different SSTs. For these reasons, the theory of passive sperm loss is the closest and most acceptable for the interpretation of "last male sperm precedence". When the female is inseminated successive times by different males and when the interval between each insemination and the subsequent one is longer, a daily loss of sperm from the SSTs occurs until the time of the new mating, making every next male fathering a greater proportion of the offspring compared to the preceding male^17^.

There is no doubt that sperm concentration in the ejaculate is considered an important factor affecting the outcome of sperm competition. If a female was inseminated by an ejaculate of a male which had a much smaller number of sperm than the one that preceded it from another male, the first male would father a greater number of the offspring outcome^7^.

In addition, sperm quality, particularly high mobility, influences the outcomes of sperm competition. When a female is inseminated by ejaculates of high and low sperm mobility from different males, the sperm cells from the first type of ejaculate fertilizes most of the ova^9^. However, this will not be the case if the sperm number in the low mobility ejaculates is significantly higher than that of the high mobility ejaculates^18^. From these observations, it appears that there are interactions between a number of factors especially sperm mobility and concentration in the ejaculates that affect the results of sperm competition. Forman et al.^19^ demonstrated that the number of sperm inseminated mainly determines the number of sperm stored in the SSTs, while sperm mobility influences the timing and rate of sperm loss from the SSTs in which low mobility sperms exit first. Therefore, when a female copulates with two males differ in sperm mobility, the sperm from the low mobility male fertilizes the first eggs^18^. Through the literature, there are some questions that still need to be answered; (1) what happens to the paternity proportions if the female is inseminated by two ejaculates of two males that are comparable in sperm number and mobility? (2) Is there another factor that may affect the results of sperm competition and may bias the paternity proportions towards a certain male?

The cryptic female choice is considered another factor that may interfere with the sperm competition outcomes. This cryptic choice enables promiscuous females to avoid inbreeding after mating by making selection against related male sperm within the reproductive system, which consequently results in improving the offspring genetic diversity^20,21^. However, the ability of the female to bias her sperm selection in favour of non-relative males disappears in the case of artificial insemination, which indicates the role that male phenotype and the act of eye-sighting during mating may play in the selection process^21^.

The duration of sperm storage in the SSTs vary from species to another. Chickens and turkeys for example can store sperm for up to 3 and 10 weeks, respectively^22^. It was reported that the number of SSTs in the uterovaginal junction determines sperm longevity in different species^23^. In the house sparrow *Passer domesticus*, shorter spermatozoa were found to have longer lifespan in the SSTs^24^. This indicates that morphometric measures influence not only sperm velocity but also sperm longevity.

It was suggested that stored sperm in the SSTs are being in a quiescent state and that they agglutinate with each other^4^. This head-to-head sperm agglutination was observed in the lumen of the SSTs in chicken^4,5^, quail^22^, and turkey^6^. It was suggested that the state of the sperm being in an agglutinated mode in the SSTs may provide a reasonable explanation of the prolonged sperm storage^22,25^. When the agglutination state is not sustained, sperm exit the SSTs. Van Krey et al.^4^ suggested that random detachment of agglutinated sperm is responsible for the gradual egress of spermatozoa into the lumen of the oviduct.

### Objectives

This experiment was conducted to study the tendency of sperm to agglutinate in motile bundles in Sharkasi and Dandarawi strains and its relationship with (1) sperm competitiveness when females are inseminated with a heterospermic pool of semen from both strains. (2) prolonging the fertility duration in both strains.

## Results

### Experiment one

**Figure 1:**
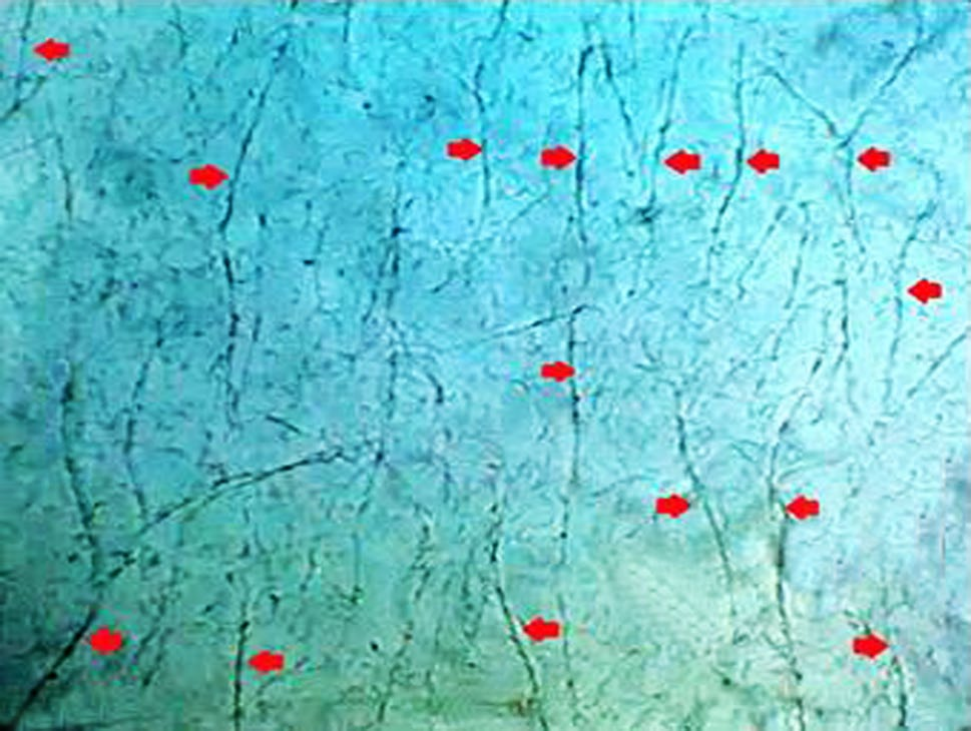
Sharkasi sperm agglutinations forming thread-like bundles in a static environment on a glass slide under phase contrast microscope. A magnification of (× 200) was used.

**Figure 2:**
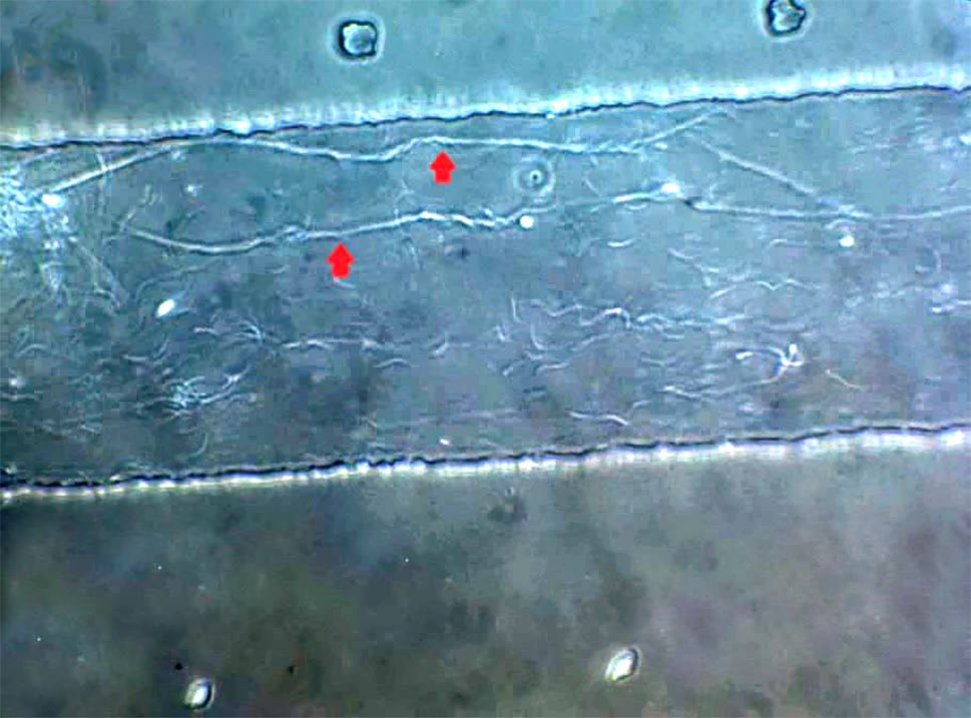
Two Sharkasi sperm thread-like bundles swimming in a dynamic environment parallel to the sidewall of a microfluidic channel of 200 µm × 20 µm dimensions (W × H) under phase contrast microscope. A magnification of (× 400) was used.

In Sharkasi chickens, when a drop of diluted semen (from individual ejaculate) was examined under a phase contrast microscope, we observed that groups of sperm (tens of cells) start to line up and aggregate within seconds post-ejaculation to form thread-like bundles (Figs. 1 and 2). A detailed description of the sperm bundles is reported by El-sherry et al.^26^. With time, the bundles grow and increase in diameter and length (Supplementary Video 1). In a static medium, the bundles started to attach to each other forming a net of threads. However, in a dynamic environment, the sperm bundles were motile and swam parallel to each other in a spiral-like movement (Supplementary Video 2). It was observed that sperm bundles increase the linear movement of the sperm. This is because lonesome sperm swim in different directions, while the bundles move progressively with or against the flowing fluid. The linear movement of the sperm bundles was assessed subjectively under the phase contrast microscope, where it was found that the bundles move parallel to each other along the walls of the microchannels of the microfluidic chip. When a flow was generated by hydrostatic pressure, the sperm bundles showed progressive spiral motility. Sperm bundles also showed high tendency to stick to the walls of the slide channels in an attempt not to be drained with the flow direction. On the other hand, fewer, shorter and thinner thread-like bundles were observed in Dandarawi samples under phase contrast microscope (Supplementary Video 3, 4).

We have examined the effect of increasing the dilution rate of semen by Lake and Ravie extender on sperm agglutination. Semen samples from both strains were extended at dilution rates of (1:1), (1:20), (1:40), (1:60), (1:80), (1:100) and (1:200). The sperm bundles In Sharkasi were thicker and longer and formed an elaborated network of bundles which were not affected by increasing the dilution level until reaching a dilution rate of 1: 40 (v: v; Supplementary Video 5). When the dilution rate increased to 1: 60 (v: v) the bundle network disappeared and only individual bundles were evident (Video 6). The motility of Sharkasi individual sperm and bundles was affected (low motility) when the dilution rate reached 1: 200 (Video 7). On the other hand, Dandarawi sperm bundles were evident but smaller in size when samples were extended until reaching a dilution rate of 1: 40 (v: v; Video 8) and died when samples were diluted 1: 80 (v: v) by Lake and Ravie diluent. (Video 9).

**Figure 3:**
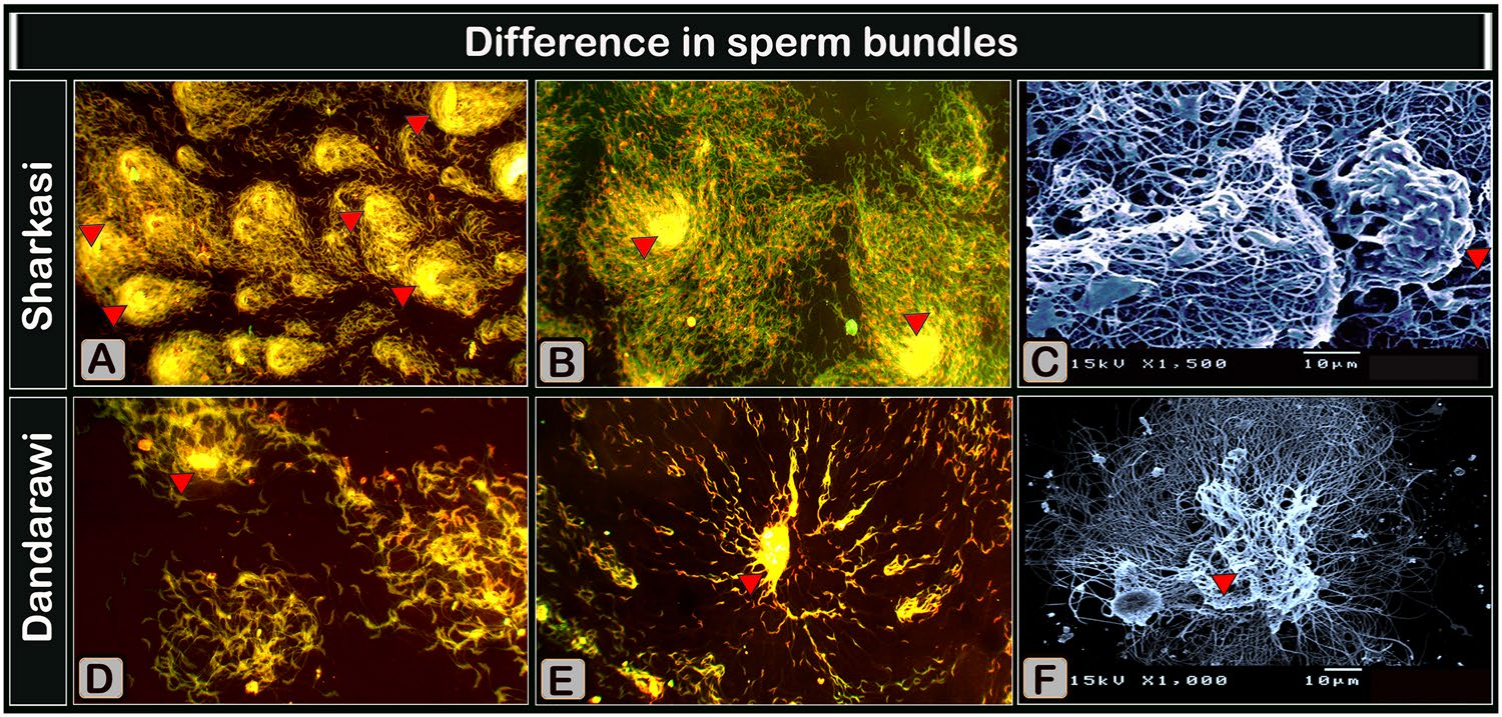
Acridine orange-stained sperm smears (**A**, Magnification × 200, **B**, Magnification × 400, **D**, Magnification × 400, **E** Magnification × 400,) and digitally colored scanning electron micrographs (**C**,**F**) images of sperm bundles. (**A**,**B**,**D**,**E**) are micrographs of sperm smear stained with Acridine orange and showing a network of adherent multiple sperm, whereas C and F are digitally colored scanning electron micrographs of sperm. (**A**,**B**) Many aggregations of Sharkasi sperm showing their high tendency of agglutination (sperm heads covered by a secretion or coat showing that sperm bundles were held by extracellular substances “agglutinating substance”, yellow color, arrowheads). (**D**,**E**) Few and small aggregations of Dandarawi sperm showing less tendency of agglutination and more scattered lonesome sperm. (**C**) Sharkasi sperm aggregates forming a network of adhered tails and heads (arrows) coated by a dense coat (agglutinating substance, arrowheads). (**F**) Dandarawi sperm aggregations with less dense agglutinating substance (arrowheads).

The differences between Sharkasi and Dandarawi sperm bundles were also confirmed by examining sperm morphology using scanning electron microscope. The images revealed enormous coating substance covering the agglutinated sperm in Sharkasi and in a far less degree in Dandarawi. Examination of sperm smears stained by Acridine orange using fluorescence microscope revealed more sperm aggregations and agglutinations in Sharakasi and more scattered sperm in Dandarawi (Fig. 3).

The obtained data of body weight, semen pH, ejaculate volume, sperm concentration, subjective sperm motility, and motility score in Sharkasi and Dandarawi roosters are presented in Table 1.

**Table 1:** Rooster body weight, semen pH, ejaculate volume, sperm concentration, subjective motility, and motility score in Sharkasi and Dandarawi strains.

	Sharkasi	Dandarawi	*p*-value
BW (g)	1964 ± 44	2025 ± 51	0.38
pH	7.13 ± 0.09	6.96 ± 0.13	0.35
Volume (µl)	244.4 ± 15.6^a^	188.2 ± 12.3^b^	0.017
Sperm Conc. (× 10^6^ /ml )	2933 ± 187^a^	2258 ± 147^b^	0.018
Motility Score	3.50 ± 0.29	3.36 ± 0.08	0.66

Means ± SEM was used to express all measurements. Ten roosters each strain were weighed. Semen samples (n = 5 per rooster) were collected over a period of 10 days and evaluated for physical characteristics.

^a,b^For main effects, means within a row without common superscripts differ significantly (*p* < 0.05).

No significant differences were found between Sharkasi and Dandarawi roosters in body weight, semen pH, subjective sperm motility, and the quality of motility. Compared to Dandarawi roosters, Sharkasi males had significantly higher ejaculate volumes, and sperm concentration/mL semen.

Data of sperm morphometry (length of entire sperm, head + midpiece, and tail) measurements and lonesome sperm velocity (VCL, VAP, VSL, and STR) in Sharkasi and Dandarawi ejaculates are presented in Table 2.

**Table 2:** Sperm morphometric measures (n = 30 sperm per rooster; length of entire sperm, head + midpiece, and tail), sperm velocity values, progressive motility % and spermatozoa with VAP > 30 μm/s % in Sharkasi and Dandarawi ejaculates (n = 5 samples per rooster).

	Sharkasi	Dandarawi	Pool StdErr	p-value
Total sperm length (μm)	99.9	99.8	1.66	0.96
Head + midpiece (μm)	22.4	21.3	0.67	0.22
Tail (μm)	77.5	78.4	1.65	0.78
VCL (μm/s)	82.07	79.84	0.82	0.06
VAP (μm/s)	42.68^a^	40.49^b^	0.65	0.02
VSL (μm/s)	31.05	29.47	0.59	0.06
STR	0.71	0.70	0.006	0.42
Progressive motility %	33.63	31.93	0.66	0.33
% of spermatozoa with VAP > 30 μm/s	46.29	43.06	0.67	0.55

Means ± SEM was used to express all measurements.

*VCL* curvilinear velocity, *VAP* average path velocity, *VSL* straight line velocity; STR = VSL/VAP.

^a,b^For main effects, means within a row without common superscripts differ significantly (*p* < 0.05).

The results showed no significant differences between Sharkasi and Dandarawi in the length of the entire sperm, head + midpiece, and tail. There was a tendency for Sharkasi lonesome sperm to show higher curvilinear (VCL; p = 0.06) and straight-line velocity (VSL; p = 0.065) than those of Dandarawi sperm. However, the differences between Sharkasi and Dandarawi spermatozoa in average path velocity (VAP) reached significance (p = 0.02). There were no significant differences in sperm straightness (SRT; p = 0.42) between Sharkasi and Dandarawi. The percentage of sperm displaying VSL higher than 30 µm/s was found to be 46.29% and 43.06% in Sharkasi and Dandarawi roosters, respectively, and the percentage of sperms showing progressive motility in Sharkasi and Dandarawi was 33.63% and 31.93%, respectively.

### Experiment two

Tables 3 and 4 shows sperm competition outcomes when Sharkasi and Dandarawi hens were artificially inseminated by semen pools.

**Table 3:** Parental identification of chicks produced by Sharkasi mothers.

	NA/NA	NA/na	Total
Number of chicks	111	25	136
Percentage	81.62%	18.38%	100%

*NA/NA* homozygous, *NA/na* heterozygous.

**Table 4:** Parental identification of chicks produced by Dnadarawi mothers.

	NA/na	na/na	Total
Number of chicks	88	42	130
Percentage	67.69%	32.31%	100%

*NA/na* heterozygous, *na/na* normal feathered.

The results revealed that 67.69% of the hatched chicks produced by Dandarawi mothers are descending from Sharkasi fathers. Similarly, Sharkasi roosters fathered most of the hatched chicks produced by Sharkasi mothers (81.6%).

**Figure 4:**
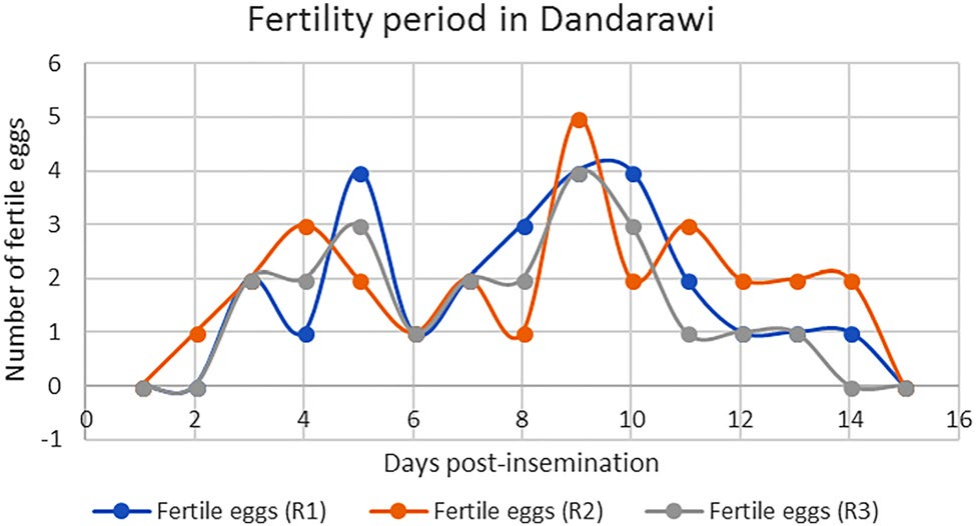
Diagram illustrating the fertility period in Dandarawi chickens (n = 10) after a single insemination. The experiment was repeated 3 times (R1, R2, R3). Dandarawi chickens continued laying fertile eggs for 14 days post insemination.

**Figure 5:**
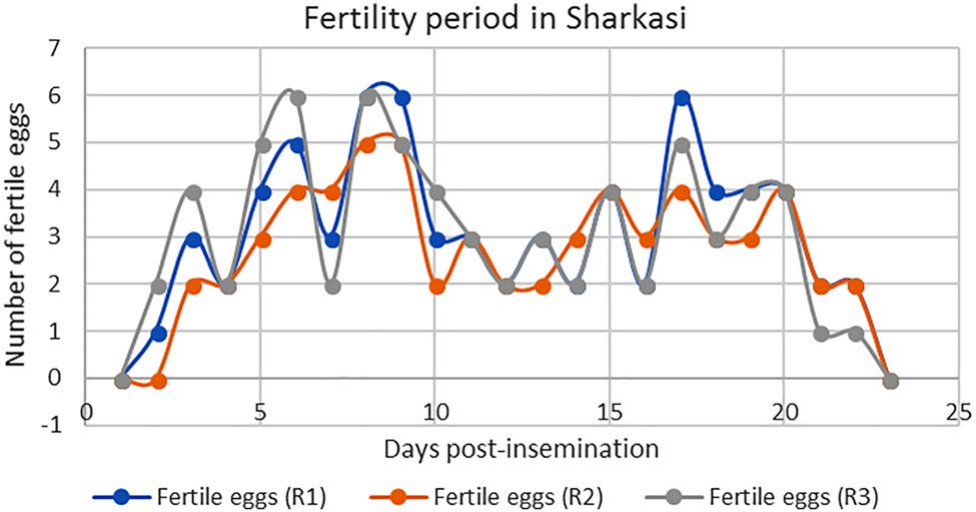
A diagram illustrating the fertility period in Sharkasi chickens (n = 10) after a single insemination. The experiment was repeated 3 times. Sharkasi chickens continued laying fertile eggs for 22 days post insemination.

Figures 4 and 5, show the maximum number of days that Sharkasi and Dandarawi hens laid fertile eggs after being artificially inseminated by Sharkasi and Dandarawi semen, respectively.

It was found that Dandarawi hens produced fertile eggs for 14 days after being artificially inseminated by Dandarawi semen. However, the fertility duration of Sharkasi hens was extended to 22 days post-insemination by Sharkasi semen.

## Discussion

### Sperm agglutination

The results of the current study showed clear differences between Sharkasi and Dandarawi chickens in the degree of sperm agglutination represented by differences in the thickness and length of the sperm bundles, which were revealed by the examination under the phase-contrast microscope and confirmed by the examination using the scanning electron microscope and fluorescence microscopy. We have previously described for the first time how the formation and movement of these bundles occur in our laboratory^26^. It was also observed that, from our previous study, once the semen is released from the male genital tract, sperm agglomerate and is sequestered from the semen plasma. In addition, we showed that the sperm bundle is formed by wrapping the sperm around one another and then sticking to each other by means of a dense substance covering parts of the sperm bundle. These bundles have distinctive characteristics as they move parallel to each other and increase the linearity of sperm movement and the number of sperm showing positive rheotaxis, and they have a high ability to stick to any stationary object around them, which increases their resistance to being swept by a high velocity flowing fluid.

In humans, while many factors contribute to male infertility, Sperm agglutination can not be neglected^27^. Although agglutination is mostly attributed to the presence of antisperm antibodies (ASAs), there are other factors such as genital tract infections and exposure to oxidative stress that can cause agglutination as well^28,29^. Since ASAs were described in 1954, It has been suggested that ASAs are responsible for autoimmune infertility based on their increased prevalence in infertile males^27^. A number of risk factors are associated with the development of ASA in males, including the breakdown of the blood-testis barrier^30^, surgical trauma^27^, pathogenic infections^31^, and prostate inflammation^32^. Antisperm antibodies can be also produced in the female reproductive system during intercourse if there is trauma to the vaginal mucous membrane^27^. Antisperm antibodies can negatively affect fertility by decreasing sperm motility and/or fertilizing capacity by agglutination which prevents sperm progression in the female reproductive tract^33^, or by impairing sperm capacitation and acrosome reaction resulting in sperm-ovum interaction failure^34,35^.

A number of studies have demonstrated that acquired immune responses influence fertilization success in birds^36^. For instance, exposure of laying hens to sperm by subcutaneous injection of sperm suspension led to an immune response (a rise in sperm antibodies in plasma) and temporal sterility for 12–67 days^37^. Also, female immune reactions due to infections or injuries in the oviduct could negatively affect sperm following mating.

Trials were conducted to understand gamete recognition and agglutination in several animals. During sea urchin fertilization, sperm adhere to eggs by a protein derived from the sperm acrosome vesicle^38^. This protein (bindin) binds the acrosome process with glycoprotein receptors on the egg vitelline layer. It was found that the sperm of sea urchins do not adhere to the vitelline layer of foreign eggs in most interspecies inseminations. It was concluded that bindin does not bind to the glycoprotein receptors from other species (species-specific^38^.

Baccetti and Afzeliu^39^ reported that the spermatozoal glycocalyx is essential for gamete recognition and agglutination. Treating fowl spermatozoa by neuraminidase resulted in modification of the glycocalyx coat by hydrolyzing the α-glycosidic bonds which in turn resulted in decreased fertility without affecting sperm vitality^5^. The researchers postulated that altering the glycocalyx by neuraminidase impaired sperm sequestration at the uterovaginal junction, resulting in a decrease in fertility. In spite of this, their observations did not exclude the possibility that neuraminidase treatment might have reduced the recognition of sperm oocytes. In another experiment, it was found that intravaginal insemination with neuraminidase-treated spermatozoa decreased fertility rates in hens, however, intramagnal inseminations by were not associated with changes in fertility in comparison to control birds^5^. These results indicate that manipulation of glycocalyx covering the sperm reduces fertility by perturbing sperm sequestration in the SSTs which increases the rate of sperm loss from the storage organ, and not by decreasing sperm-egg recognition.

On the other hand, in some animals and insects, it was found that the accumulation and adhesion of sperm to form mobile sperm bundles does not negatively affect fertility, but on the contrary, plays a beneficial role as it helps to increase the competitiveness of sperm in different ways. For example, in monotremes, spermatozoa form bundles of about 100 cells arranged roughly parallel to one another as they leave the initial segment of the epididymis and this was found to greatly enhance the forward velocity of the spermatozoa^40^. The bundle formation involves the binding of sperm heads together. An epididymal secretory protein, acidic cysteine-rich osteonectin (SPARK), that is responsible for bundle formation was identified^40,41^. In addition, the dispersal of these bundles was associated with the loss of this protein^40^. Another example was found in the common wood mouse, *Apodemus sylvaticus* where spermatozoa undergo a unique morphological transformation concomitant with the adhesion of the apical hook's concave surface to the flagellum or other spermatozoa's apical hooks that results in the organization of hundreds of cells in distinct aggregates, supporting rapid progressive motility of sperm^42^.

In this study, it was observed that bundles of sperm formed (tens of cells) immediately after ejaculation in both strains under study, with a marked difference in the degree of agglutination of sperm, as it appears clearly in Sharkasi chickens and to a lesser extent in Dandarawi. These bundles grow in thickness and length over time and branch out to form a network of bundles when in a static environment. They are also mobile and swim parallel to each other if they are in a dynamic environment.

It was found that these bundles were not affected by extending semen with Lake and Ravie diluent and increasing the dilution rate up to a rate of 1:40 in both strains. It was observed that the sperm bundles in Sharkasi chicken intertwine with each other to form a complex network of thick bundles, which was absent in the case of the Dandarawi chicken, whose sperm contained only small individual bundles. From these results, it can be concluded that the agglutination of the sperm and the formation of these bundles are not attributed to the concentration of sperm and the volume of the ejaculate. When the dilution rate was increased to 1:60, the network of bundles disappeared and only individual bundles of sperm remained in the Sharkasi strain. Sperm motility began to be negatively affected when the dilution ratio reached 1:200. In Dandarawi chickens, it was observed that sperm motility was affected at a dilution rate of 1:60 and died when it reached 1:80. This difference observed in sperm tolerance to increasing dilution rate may be attributed to either the strain (genotype) or to the substance responsible for sperm agglutination. Using the scanning electron microscope and by examining Acridine-stained semen smears, a coat of agglutinating substance was found densely covering the sperm bundles in Sharkasi chickens. It can be suggested that sperm bundles under study did not form as a result of the adherence to antisperm antibodies for several reasons; the first of which is that fertility was not adversely affected. Also, the results of sperm competition were always in favor of the Sharkasi roosters, whose sperm tended to agglutinate to a greater extent than that of Dandarawi, which had to be the opposite in the case of adhesion to the antisperm antibodies.

Significant differences in ejaculate volume and sperm count per mL between Sharkasi and Dandarawi chickens were observed. The ejaculate volume of Sharkasi roosters averaged 244 µL which was significantly higher (p = 0.017) than that of Dandarawi males by 56 µL. The sperm count in 1 mL of Sharkasi semen was higher than that of Dandarawi by 29.9%.

These differences in ejaculate volume and sperm count per mL between Sharkasi and Dandarawi roosters can be attributed to genotype. Measuring semen volume and sperm concentration is an important part of semen evaluation process, primarily because this information is required to calculate the appropriate rate of dilution in order to obtain adequate number of spermatozoa (100 million sperm) per insemination. Although, high or low volumes of semen are neither detrimental nor beneficial to fertilizing capacity, it is desirable to obtain larger volumes of semen from the fewest number of males for artificial insemination purposes^43^. Peters et al.^44^ evaluated the semen quality in 6 exotic and indigenous chicken strains and reported that the highest sperm concentration was found in the naked neck cocks compared to frizzled and normally feathered roosters. The variations in sperm concentration among frizzle, naked neck and normally feathered roosters were attributed to their genotype^45,46^.

A significant body of evidence has shown that morphometric measures of spermatozoa are related to their velocity. For example, it was assumed that longer sperm swim faster, because they contain more mitochondria in the midpiece producing more ATP, which provides the flagellum with higher energy needed for oscillatory movement. This relationship between sperm length and velocity was reported in roosters, Iberian deer, zebra finch, cichlid fishes and in passerine birds^47–51^.

In the current study, no significant differences were observed in sperm morphometric measurements between Sharkasi and Dandarawi. These results agree with those of Sayed et al.^52^, and indicate that any differences in sperm swimming velocity in this experiment cannot be attributed to differences in sperm morphometric measures between Sharkasi and Dandarawi. Although, the variation in the mean values of sperm VAP between Sharkasi (VAP = 42.68) and Dandarawi (VAP = 40.49) was small, the difference was statistically significant (P < 0.05). While, no significant differences in VSL and VCL were observed between Sharkasi and Dandarawi spermatozoa.

Sperm mobility is defined as the net forward movement of sperm against resistance at body temperature^53^. Forman and Mclean^54^ assayed chicken sperm mobility against Accudenz solution at a temperature of 41 °C. It was reported that sperm straight line velocity VSL should exceed 30 µm/s in order to penetrate the Accudenz solution^13^. It can be concluded from these reports that mobile sperm must show progressive motility and a minimum VSL of > 30 µm/s.

In the current study, based on the percentages of spermatozoa showing progressive motility (33.63% and 31.93%) and VSL higher than 30 µm/s (46.29% and 43.06%) in Sharkasi and Dandarawi chickens, respectively, it can be assumed that both chicken strains had comparable sperm mobility.

### Sperm competition

The paternity identification was determined for 136 hatchlings produced by Sharkasi mothers. The Sharkasi roosters fathered 111/136 (81.62%) of the offspring, while Dandarawi fathered 25/136 (18.38%) chicks. On the other hand, the results of paternity identification for 130 hatchlings produced by Dandarawi mothers have shown that Sharkasi roosters fathered 88/130 (67.69%) of the offspring, while Dandarawi males fathered 42/130 (32.31%) chicks.

It is well established that semen quality is an important determinant of fertility^44^. Sperm number in the ejaculate influence sperm competition outcomes^7^. Therefore, in the current study, the hens have been inseminated by a semen pool containing an equal number of sperms from Sharkasi and Dandarawi roosters to negate any effect of sperm concentration on sperm competition results.

Cardullo and Baltz^47^ showed that sperm length is associated with its ability to compete because longer sperms contain more mitochondria in the midpiece and consequently, producing more ATP which is utilized during swimming increasing the velocity. Other studies reported that sperms with small heads in relation to their flagellum showed higher swimming velocity^24,49^. The current results showed no differences between Sharkasi and Dandarawi roosters in sperm morphometric measures. Therefore, sperm morphometric measures are not the determinant factor justifying the variation seen in sperm competition results.

Sperm that have higher mobility reach the SSTs faster and fill more vacant spaces than sperm with lower mobility^55^. Although we did not measure sperm mobility according to the method of Forman^53^, we assessed other measurements related to sperm mobility which are the percentage of sperms that show progressive movement and the percentage of sperms swimming at a VSL greater than 30 µm/s. It was found that both measures were comparable in Sharkasi and Dandarawi semen. This might indicate that Sharkasi and Dandarawi sperm have nearly similar mobility.

Sperm competition happens when spermatozoa of different roosters compete to reach and fertilize the ova^56,57^. This competition is based on the way the female stores the spermatozoa and biases sperm selection towards a particular rooster^58^.When competing with more than one male, females generate post insemination intersexual selection, which is known as “cryptic female choice”^59^. Differential sperm ejection has been suggested as a mechanism of cryptic female choice in a diverse group of organisms^58,60^. Females choose genetically dissimilar males to increase the genetic diversity of their offspring instead of increasing inbreeding that leads to reduced fitness^1,61–64^. This choice may lead to the improvement of the offspring by biasing sperm use in response to the females' similarity to a male^65,66^. In the current study, in both cases, when Sharkasi and Dandarawi females were inseminated by the semen pool, Sharkasi roosters showed superiority compared to Dandarawi males in dominating the fertility results. This dominance was much pronounced when Sharkasi females were used. In addition, hens must mate with roosters naturally in order to bias its selection towards specific males. In other words, the ability of females to select spermatozoa of particular males is absent when they were artificially inseminated.

Here, we attribute the differences seen in the sperm competition results to the extraordinary sperm agglutination behavior observed in Sharkasi and in a far less degree in Dandarawi chickens. Earlier studies have shown that sperm agglutinate head-to-head and remain in a quiescent state during their storage in the SSTs of chicken^4,5^, quail^22^ and turkey^6^. This sperm agglutinated state may explain sperm storage for a prolonged period in the SSTs^22,25^. The gradual egress of spermatozoa into the oviduct lumen is thought to be caused by random detachment of agglutinated sperm^4^. Therefore, we suggest that the agglutination behavior of Sharkasi sperm gives them superiority over Dandarawi sperm when present in a competitive situation to fertilize the ova because sperm are lost from the SSTs when the agglutination state is not sustained.. El-sherry et al.^26^ reported that sperm agglutination in motile bundles increases the number of sperm showing positive rheotaxis which is suggested to benefit sperm during the vaginal selection process (passage of sperm through the vagina) and the SSts uptake (sperm penetration into SSTs). According to this assumption, more sperm from Sharkasi will occupy vacant areas of the SSTs compared to Dandarawi sperm. Moreover, irrespective of this assumption, the higher tendency of Sharkasi sperm to agglutinate inside the SSTs keeps their residence for longer periods which in turn increases their chances to fertilize more ova than Dandarawi sperm. In other words^26^, lonesome sperm is suggested to exit from the SSTs first (followed by those detached from agglutinated sperm bundles. So, lonesome sperm (few Sharkasi and most of Dandarawi sperm) is suggested to egress the SSTs and compete to fertilize the first egg, then the sperm detached from agglutinated bundles remaining in the SSTs (few Dandarawi and most of Sharkasi sperm) compete to fertilize the subsequent eggs which gives higher opportunity to Sharkasi sperm to fertilize more ova.

In the current study, the results showed that Sharkasi chickens have longer fertility period (22 days) compared to Dandarawi chickens (14 days). Unlike mammals, the SSTs store spermatozoa for a period of 2–15 weeks in domestic birds which varies according to the species^22,67^. The fertility period in both chickens and turkeys depends on their ability to store and maintain sperm in the SSTs of the utero-vaginal junction. This period is thought to be related to the numbers of SSTs. The fertility period in chickens ranges from 2 to 3 weeks, and the number of SSTs in the oviduct was estimated to be around 5000. On the other hand, turkey's oviduct contains around 30,566 SSTs and the fertility period extends to 10–15 weeks. This might indicate that the number of SSTs affects sperm physiology^25,67^. This big difference in the number of SSTs between turkeys and chickens might be attributed to either the species or to the difference in body size. In the current study, there were no significant differences in body size between Sharkasi and Dandarawi hens to suggest any differences in the number of SSTs that can justify the differences in the fertility period between the two strains. One of the factors that influence sperm longevity in the SSTs is sperm length. Stockley et al.^68^ found that sperm longevity decreases with increases in sperm length across several species of fish. There is a negative correlation between sperm longevity and sperm size^69^. On the other hand, longer sperms of Sand Martin *Riparia Riparia* were found to have longer life but slow swimming velocity compared to shorter sperm^24,70,71^. Because we did not find any significant differences in sperm morphometric measures between Sharkasi and Dandarawi chickens, sperm morphometry is excluded from the factors influencing the sperm longevity in the current study.

Although sperm agglutination is considered one of the reasons causing infertility in humans^2^, it is likely being advantageous in chickens, because the agglutinated sperm bundles are motile, thus sperm are capable of reaching the uterovaginal junction and being stored inside the lumen of the SSTs. Our results showed that the agglutinated sperm are covered with a copious substance which is suggested to be responsible for adhering sperm together and clinging sperm to any adjacent surface. Therefore, we assume that Sharkasi spermatozoa stick to the walls of the SSTs lumen which make them remain for a longer period. Since Sharkasi sperm showed higher tendency to agglutinate and form thicker and longer sperm bundles than those of Dandarawi, we suggest that Sharkasi sperm bundles remain in the SSTs for longer time because they will spend more time to completely disperse compared to those of Dandarawi. Our results strengthen previous observations of^4–6,22^, Bakst and Bauchan^6^ who reported that spermatozoa are stored in a state of head-to-head sperm agglutination in the lumen of the SSTs in chicken, quail and turkey and when this state of agglutination is not sustained by random detachment of sperm, they gradually exit from SSTs^4^.

This study is the first to discuss the tendency of sperm to agglutinate as a factor affecting the outcomes of sperm competition and the length of the fertility period in chickens. The study showed that the tendency of spermatozoa to agglutinate differs from one strain to another. In addition, it most likely increases the chances of sperm to remain for a longer period within the sperm nests (SSTs) and increases their competitiveness to fertilize the ova. Further studies are required to test the normal distribution of this phenomenon in chicken flocks, find a method to measure the sperm tendency to agglutinate rather than using descriptive observations, and determine the type of substance responsible for agglutination, which is believed to be a protein and study whether it is related to sperm tolerance to high levels of dilutions and whether it is able to enhance the vitality of sperm in liquid stored semen which would improve the application of artificial insemination and fertility rates.

## Materials and methods

### Ethical approval

The research was carried out in conformity with the applicable standards and regulations. The National Ethics Committee of Assiut University and veterinary authorities in Assiut Province, Egypt, both approved. Egypt’s Committee also approved the study for the Use and Care of Experimental Animals and the Faculty of Veterinary Medicine and Agriculture in Assiut. All methods were performed in accordance with the relevant guidelines and regulations".

### ARRIVE guidelines

The study was carried out in compliance with the Animals in Research: Reporting In Vivo Experiments (ARRIVE) guidelines^72^.

Thirty-week-old healthy mature male (n = 10) and female (n = 20) from both naked neck Sharkasi (homozygous dominant; Na Na) and Dandarawi (normal feathered) chickens were selected from the basic flocks of Poultry Research Farm, Faculty of Agriculture, Assiut University Assiut Governorate, Egypt, according to their response to semen collection, semen quality attributes, and egg-laying rate and were used in this study. All birds were housed in individual cages (30 × 40 × 40 cm), exposed to a lighting program of 16 h Light:8 h Dark, fed a commercial diet containing 160 g crude protein, 2800 kcal metabolizable energy, 35 g calcium, and 5 g available phosphorus per kg diet and provided with fresh water ad libitum.

### Experiment one

Experiment one aimed to compare between Sharkasi and Dandarawi semen physical characteristics, sperm morphometry, and sperm velocity. A total number of ten roosters from each strain were used in this experiment.

### Semen collection and analysis

Semen was collected from all roosters five times over a period of ten days, using the method of abdominal massage according to Burrows and Quinn^73^, to evaluate ejaculate volume, color, pH, sperm concentration, subjective motility and motility score. Any ejaculates contaminated with blood or feces was discarded. Sperm morphometric measures (entire sperm, sperm head + midpiece, and tail length), and sperm velocity were evaluated using computer assisted sperm analysis (CASA) plugin for Image-J software.

### Evaluation of semen physical characteristics

Semen volume was measured by a graduated collection tube. Sperm concentration in five ejaculates per rooster in each strain was measured using a hemocytometer (Neubauer chamber). Semen pH was measured using a digital pH meter (ADWA, AD11 Waterproof pH-TEMP Pocket Tester, 6726 Szeged–Hungary). The quality of sperm motility was scored on a scale of 0–5 as described by Santiago-Moreno et al.^74^.

### Sperm morphometry

A number of 30 samples of semen were collected from each strain (3 samples per rooster) and diluted to 1:200 using Lake and Ravie diluent. The diluted samples were fixed by adding formalin to a final concentration of 5%. A drop of formalin-fixed semen was deposited on a glass slide and a smear was made and air-dried. The slide was then rinsed with distilled water^19^. Photographs of spermatozoa were taken with a digital color video camera (Sony- CCD-IRIS/ RGB) mounted on a microscope at × 400 magnification. From these pictures, ten intact spermatozoa from each slide were selected (no broken tail, midpiece correctly coiled around the flagellum) and the entire sperm, head + midpiece and flagellum lengths for each sperm were estimated using the software Image-J.

### Sperm kinetics

Fifty semen samples (Five samples per rooster) from each strain were analyzed over the experimental period to assess sperm velocity. Sperm swimming velocity were assessed using a new CASA plugin for Image-J, an image processing software provided by National Institution of Health (http://imagej.nih.gov/ij/), where various features were added to the original plugin to enhance sperm tracking^75^.

A drop of diluted semen (1: 40 v/v Lake and Ravie diluent) was placed on a microscope slide, and covered with a glass cover, then placed on a microscope for examination. A magnification of (400x) was used and several fields were examined to evaluate motility %, curvilinear velocity (μm/sec; VCL), average path velocity (μm/sec; VAP), straight line velocity (μm/sec; VSL) and straightness (STR = VSL/VAP). Videos of sperm movement were recorded using Tucsen ISH 1000 camera at 30 frames/second mounted on Optika XDS-3 inverted microscope with phase contrast. A minimum 3 fields and 500 sperm tracks were examined. Spermatozoa with VAP < 10 µm/s and VSL < 5 µm/s are considered immotile. The percentage of spermatozoa demonstrating progressive motility was calculated as the number of spermatozoa exceeding 20 µm/s VAP and 80% STR divided by the number of motile spermatozoa.

### Sperm tendency to agglutinate

The tendency of spermatozoa to agglutinate in static and dynamic environments was assessed by recording videos of diluted semen 1: 1 (v: v) when placed on a glass slide and covered by a cover slip (static environment) or loaded into a microfluidic slide when a flowing fluid was generated by hydrostatic pressure (dynamic environment^75^) under a phase contrast microscope (Optika XDS-3 inverted microscope with phase contrast). The effect of increasing the dilution rate of semen by Lake and Ravie extender on sperm agglutination was studied. Semen samples from both strains were extended at dilution rates of 1:1, 1:20, 1:40, 1:60, 1:80, 1:100 and 1:200 (v:v).

### Semen smears stained with Acridine orange stain and scanning electron microscopy

Semen smears were prepared and stained with Acridine orange stain and analyzed using a microscope (model Letiz DM 2500) with external fluorescent unit (Leica EL 6000). The morphology of the sperm and the tendency to agglutinate in bundles were also assessed using scanning electron microscope.

### Acridine orange stain^26,76^

Sperm samples (n = 6 for each stains) were collected, diluted, Semen samples from both strains were extended at dilution rates of 1:40. A drop of diluted semen (1: 40 v/v Lake and Ravie diluent) was placed on a microscope slide, Distilled water, acridine orange, and glacial acetic acid make up the stain. Preparation of reagents: A 50 mg acridine orange stock solution was made in 10 mL distilled water and kept in the refrigerator. Combine 1 mL acridine orange stock solution and 0.5 mL glacial acetic acid in 50 mL distilled water to generate a working solution. Method of staining: On a clean slide, apply a drop of diluted semen and allow it to air dry.The slide was then methanol-fixed and dried once more. After that, it's put in a trough with an acridine orange staining solution (i.e., 0.01 percent). After staining for 2 min, the slides were gently cleaned and dried before being viewed. The slides examined using a microscope (model Letiz DM 2500 with external fluorescent units), (Leica EL 6000).

### Scanning electron microscopy

Sperm samples (n = 6 for each strains) were collected, diluted (Semen samples from both strains were extended at dilution rates of 1:40) and kept in Karnovsky fixative were kept in Karnovsky fixative^77^ in Eppendorf tubes for scanning electron microscopy investigation after semen collection and dilution.

A scanning electron microscope (SEM) was used to observe the formation of sperm bundles. The following procedures were followed for sample preparation: Each sample was fixed for 4 h at 4 °C in Karnovsky fixative^77^. The samples were then centrifuged and rinsed in the same fixation buffer five times before being post fixed in 1 percent osmic acid in 0.1 M Na phosphate buffer for two hours at room temperature. They were washed with 0.1 M Na-phosphate buffer 15 times. The samples were dried in ascending grades of ethanol alcohol with concentrations of 50, 70, 90, and 100 percent (30 min in each concentrations Each sample was distributed on an aluminum foil boat, fastened, and then air-dried. Finally, the samples were coated with gold using a JEOL1100 E ion sputtering device and examined at KV10 using a JEOL scanning electron microscope (JSM5400 LV). Each sample was distributed on an aluminum foil boat, fastened, and then air-dried. Finally, the samples were coated with gold using a JEOL1100 E ion sputtering device and examined at KV10 using a JEOL scanning electron microscope (JSM5400 LV).

### Digital coloring of scanning electron microscopic images

We used Photoshop to digitally colour the scanning electron microscopic images. The methodology has already been used by several authors^78–82^.

### Experiment two

This experiment aimed to assess: (1) the competition between Sharkasi and Dandarawi spermatozoa on fertilizing the ova. (2) To assess the fertility duration (certain period a hen can produce fertile eggs after a single insemination) in both chicken strains. (3) To use the results obtained from experiment 1 to justify those obtained from experiment 2.

**Figure 6:**
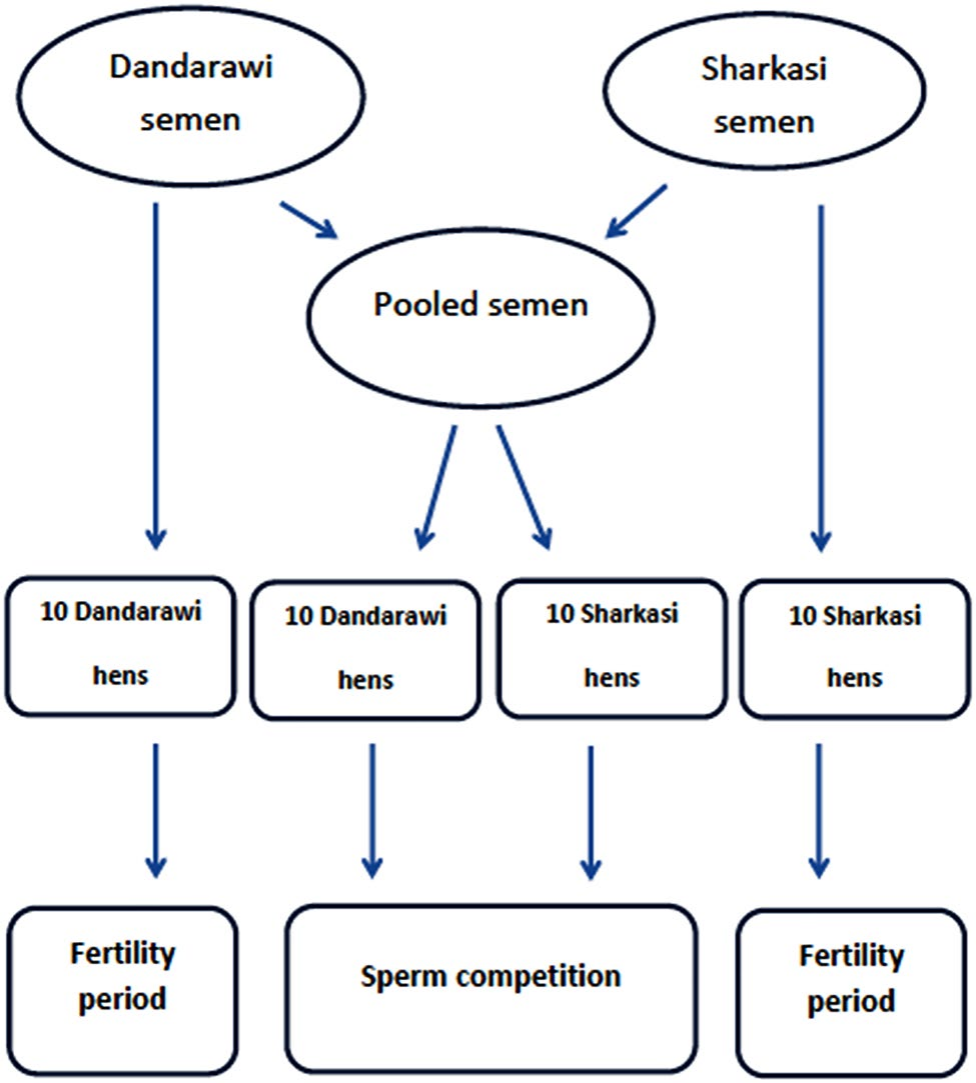
A diagram demonstrating the experiment design. For assessing sperm competition, Dandarawi and Sharkasi hens were inseminated 10 consecutive times (with 3 days intervals between each two consecutive inseminations) by semen-pooled ejaculates collected from both breeds so that each inseminated semen pool contained an equal number of sperm from both strains. To evaluate fertility duration, Dandarawi and Sharkasi hens were inseminated using semen collected from roosters of the same breed. Eggs were collected for a period of 28 days and set into the incubator.

The experiment design is shown in Fig. 6. In this experiment, 10 males and 20 females from each strain were used to assess sperm competition and fertility duration. For assessing sperm competition, ejaculates collected from Sharkasi and Dandarawi roosters were mixed in which the resultant pool had equal number of spermatozoa from both strains. Ten females from each strain were artificially inseminated using the semen pool (100 × 10^6^ cells/hen). Ten consecutive intravaginal artificial inseminations were performed (once every three days) and the laid eggs were collected the day after the first insemination and for duration of 30 days. Fertile eggs were marked and set into the incubator (38 ± 0.1 °C; 55–60% relative humidity) until hatch. Hatchlings were recognized from their phenotypes as descending from Sharkasi or Dandarawi fathers.

To evaluate the fertility duration, ten hens from each strain were artificially inseminated by semen collected from males of the same strain. Laid eggs were collected the day after the artificial insemination for a period of 4 weeks. The date of oviposition was written by a pencil on the eggshell then the eggs were set into the incubator. This process was repeated three times to find out the last day at which a hen can produce a fertile egg after one insemination.

### Paternity identification

**Figure 7:**
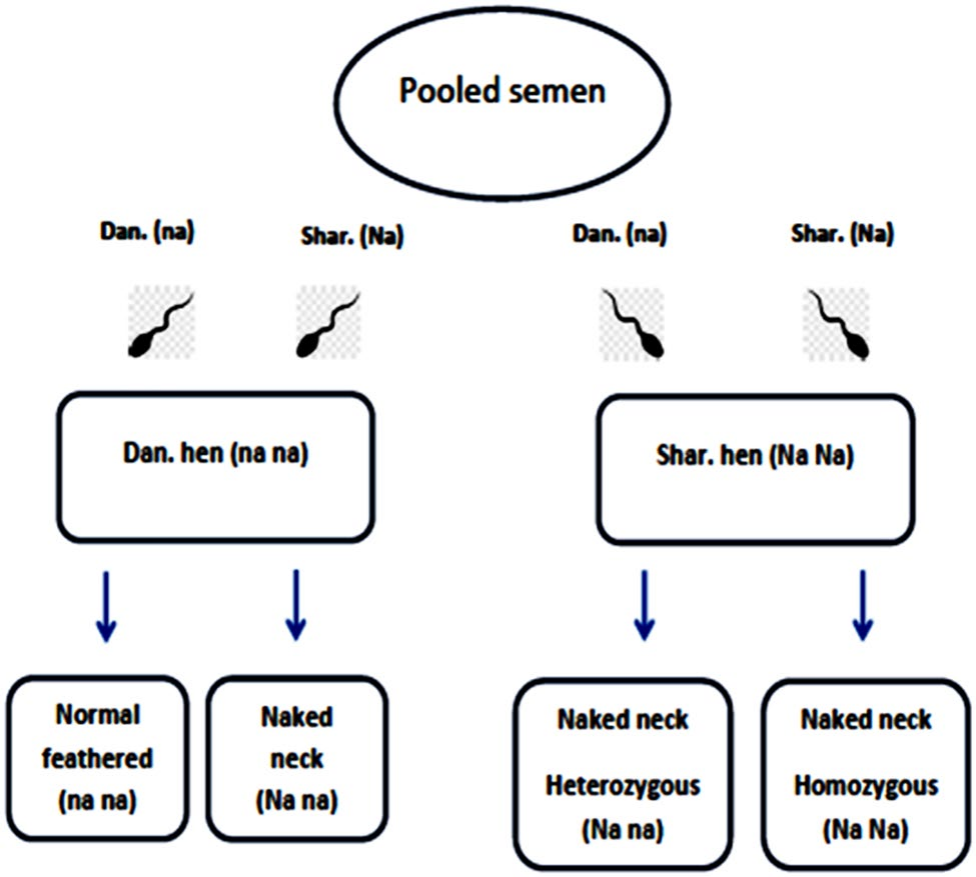
A diagram showing the method used to identify the outcome of sperm competition depending on the inheritance of naked neck gene and the hatchling phenotype.

To find out whether the hatchlings are descending from Sharkasi or Dandarawi fathers, we relied on the inheritance of the naked neck gene found in Sharkasi chickens (see Fig. 7).

The naked neck gene is responsible for reducing feather cover on the neck and vent. This trait is controlled by incompletely dominant allele (Na) which consequently is reflected on the phenotype of the birds.

Naked neck Sharkasi chickens are having three genotypes; (1) homozygous dominant (Na/Na) which is characterized by a reduction in feather mass by 33 and 41% in female and male, respectively, compared to normal feathering chickens. (2) Heterozygous (Na/na) which is having 22 and 27% less feather in female and male, respectively. (3) homozygous recessive (na/na) which is a normal feathered bird^83^. Dandarawi chickens are normal feathered birds (na/na).

### Statistical analysis

The data obtained were tested for normality distribution using Kolmogorov–Smirnov test and the Shapiro–Wilk test, the results showed that data were normally distributed, and no data transformation was needed. Subsequently, the data analyzed by ANOVA using the GLM procedure of SAS (SAS institute^84^) to compare between two different breeds as a fixed effect and the experimental error were considered as random effect. According to data descriptive measures the two different breeds have homoscedastic variance. Duncan’s multiple-range test^85^ was used to compare between different means and the differences were considered significant at the level of P < 0.05.

The following model was used for analysis of variance:

$${\text{Y}}_{{{\text{ij}}}} = \upmu + {\text{S}}_{{\text{i}}} + {\text{E}}_{{{\text{ij}}}}$$

where: Y_ij_ = observation, µ = overall mean, S_i_ = breed effect, E_ij_ = experimental errors.

## Supplementary Information

Supplementary Video 1.

Supplementary Video 2.

Supplementary Video 3.

Supplementary Video 4.

Supplementary Video 5.

Supplementary Video 6.

Supplementary Video 7.

Supplementary Video 8.

Supplementary Video 9.

Supplementary Legends.

## Data Availability

All data created or analyzed during this investigation are included in this published paper and are available on request from the corresponding author.

## References

[1] Charlesworth D, Charlesworth B (1987). Inbreeding depression and its evolutionary consequences. Annu. Rev. Ecol. Syst..

[2] Glass RH, Vaidya RA (1970). Sperm-agglutinating antibodies in infertile women. Fertil. Steril..

[3] Johnston SD, Jones RC (2016). Formation and dissociation of sperm bundles in monotremes. Biol. Reprod..

[4] Van Krey H, Balander R, Compton M (1981). Storage and evacuation of spermatozoa from the uterovaginal sperm-host glands in domestic fowl. Poult. Sci..

[5] Froman DP, Engel HN (1989). Alteration of the spermatozoal glycocalyx and its effect on duration of fertility in the fowl (*Gallus domesticus*). Biol. Reprod..

[6] Bakst MR, Bauchan G (2015). Apical blebs on sperm storage tubule epithelial cell microvilli: Their release and interaction with resident sperm in the turkey hen oviduct. Theriogenology.

[7] Birkhead TR (1998). Sperm competition in birds. Rev. Reprod..

[8] Choe JC (2019). Encyclopedia of Animal Behavior.

[9] Birkhead T, Martinez J, Burke T, Froman D (1999). Sperm mobility determines the outcome of sperm competition in the domestic fowl. Proc. R. Soc. Lond. Ser. B.

[10] Donoghue AM (1999). Paternity efficiency in turkey differs extensively after heterospermic insemination. J. Appl. Poultry Res..

[11] Martin P, Reimers T, Lodge J, Dziuk P (1974). The effect of ratios and numbers of spermatozoa mixed from two males on proportions of offspring. Reproduction.

[12] Donoghue A (2003). Field testing the influence of sperm competition based on sperm mobility in breeder turkey toms. Br. Poult. Sci..

[13] Froman DP (2007). Sperm motility in birds: Insights from fowl sperm. Soc. Reprod. Fertil. Suppl..

[14] Birkhead TR, Moller AP (1992). Sperm Competition in Birds. Evolutionary Causes and Consequences.

[15] Compton M, Van Krey H, Siegel P (1978). The filling and emptying of the uterovaginal sperm-host glands in the domestic hen. Poult. Sci..

[16] King L, Brillard J, Garrett W, Bakst M, Donoghue A (2002). Segregation of spermatozoa within sperm storage tubules of fowl and turkey hens. Reproduction.

[17] Pizzari T (2007). Post-insemination sexual selection in birds. Soc. Reprod. Fertil. Suppl..

[18] Pizzari T, Worley K, Burke T, Froman DP (2008). Sperm competition dynamics: Ejaculate fertilising efficiency changes differentially with time. BMC Evol. Biol..

[19] Froman DP, Pizzari T, Feltmann AJ, Castillo-Juarez H, Birkhead TR (2002). Sperm mobility: Mechanisms of fertilizing efficiency, genetic variation and phenotypic relationship with male status in the domestic fowl, Gallus gallus domesticus. Proc. R. Soc. Lond. Ser. B.

[20] Gillingham MA (2009). Cryptic preference for MHC-dissimilar females in male red junglefowl, Gallus gallus. Proc. R. Soc. B.

[21] Løvlie H, Gillingham MA, Worley K, Pizzari T, Richardson DS (2013). Cryptic female choice favours sperm from major histocompatibility complex-dissimilar males. Proc. R. Soc. B.

[22] Bakst MR, Wishart G, Brillard J-P (1994). Oviducal sperm selection, transport, and storage in poultry. Poult. sci. rev.

[23] Bakst M (2010). Comparisons of sperm storage tubule distribution and number in 4 strains of mature broiler breeders and in turkey hens before and after the onset of photostimulation1. Poult. Sci..

[24] Helfenstein F, Podevin M, Richner H (2010). Sperm morphology, swimming velocity, and longevity in the house sparrow Passer domesticus. Behav. Ecol. Sociobiol..

[25] Ito T (2011). Progesterone is a sperm-releasing factor from the sperm-storage tubules in birds. Endocrinology.

[26] El-Sherry TM, Abd-Elhafeez HH, Sayed M (2022). New insights into sperm rheotaxis, agglutination and bundle formation in Sharkasi chickens based on an in vitro study. Sci. Rep..

[27] Berger GK, Smith-Harrison LI, Sandlow JI (2019). Sperm agglutination: Prevalence and contributory factors. Andrologia.

[28] Hsieh T-C, Shin P (2012). Male Infertility.

[29] Marshburn PB, Kutteh WH (1994). The role of antisperm antibodies in infertility. Fertil. Steril..

[30] Cheng CY, Mruk DD (2012). The blood-testis barrier and its implications for male contraception. Pharmacol. Rev..

[31] Thaper D, Prabha V (2018). Molecular mimicry: An explanation for autoimmune diseases and infertility. Scand. J. Immunol..

[32] Kortebani G, Gonzales G, Barrera C, Mazzolli A (1992). Leucocyte populations in semen and male accessory gland function: Relationship with antisperm antibodies and seminal quality. Andrologia.

[33] Turek PJ (1997). Immunopathology and Infertility. Infertility in the Male.

[34] Srivastava P, Sheikhnejad RG, Fayrer-Hosken R, Malter H, Brackett B (1986). Inhibition of fertilization of the rabbit ova in vitro by the antibody to the inner acrosomal membrane of rabbit spermatozoa. J. Exp. Zool..

[35] Shibahara H, Burkman LJ, Isojima S, Alexander NJ (1993). Effects of sperm-immobilizing antibodies on sperm-zona pellucida tight binding. Fertil. Steril..

[36] Burke W, Yu WC (1979). Infertility in the Turkey: I. Effects of anti-sperm immune globulins on fertilizing ability of Turkey Spermatozoa. Poult. Sci..

[37] McCartney J (1923). Studies on the mechanism of sterilization of the female by spermotoxin. Am. J. Physiol..

[38] Glabe CG, Vacquier VD (1977). Species specific agglutination of eggs by bindin isolated from sea urchin sperm. Nature.

[39] Baccetti B, Afzelius BA (1976). The biology of the sperm cell. Monogr. Dev. Biol..

[40] Nixon B (2016). Formation and dissociation of sperm bundles in monotremes. Biol. Reprod..

[41] Grützner F, Nixon B, Jones R (2008). Reproductive biology in egg-laying mammals. Sex. Dev..

[42] Moore H, Dvorakova K, Jenkins N, Breed W (2002). Exceptional sperm cooperation in the wood mouse. Nature.

[43] Etches RJ (1998). Reproduction in Poultry.

[44] Peters S (2008). Semen quality traits of seven strains of chickens raised in the humid tropics. Int. J. Poult. Sci..

[45] Ajayi F, Agaviezor B, Ajuogu P, Harcourt P (2011). Semen characteristics of three strains of local cocks in the humid tropical environment of Nigeria. Int. J. Anim. Vet. Adv.

[46] Nwachukwu E, Ibe S, Amadi C (2006). Effect of genotype and frequency of semen collection on semen characteristics of local chicken cocks. J. Anim. Vet. Adv..

[47] Cardullo RA, Baltz JM (1991). Metabolic regulation in mammalian sperm: Mitochondrial volume determines sperm length and flagellar beat frequency. Cell Motil. Cytoskelet..

[48] Malo AF (2006). Sperm design and sperm function. Biol. Lett..

[49] Humphries S, Evans JP, Simmons LW (2008). Sperm competition: Linking form to function. BMC Evol. Biol..

[50] Fitzpatrick JL (2009). Female promiscuity promotes the evolution of faster sperm in cichlid fishes. Proc. Natl. Acad. Sci. USA.

[51] Lüpold S, Calhim S, Immler S, Birkhead TR (2009). Sperm morphology and sperm velocity in passerine birds. Proc. R. Soc. B.

[52] Sayed M, Abouelezz F, Abdel-Wahab AA (2017). Analysis of sperm motility, velocity and morphometry of three Egyptian indigenous chicken strains. Egypt. Poult. Sci. J..

[53] Froman D (2006). Application of the sperm mobility assay to primary broiler breeder stock. J. Appl. Poult. Res..

[54] Froman D, McLean D (1996). Objective measurement of sperm motility based upon sperm penetration of Accudenz®. Poult. Sci..

[55] Holsberger DR, Donoghue A, Froman D, Ottinger M (1998). Assessment of ejaculate quality and sperm characteristics in turkeys: Sperm mobility phenotype is independent of time. Poult. Sci..

[56] Parker GA (1970). Sperm competition and its evolutionary consequences in the insects. Biol. Rev..

[57] Pizzari T, Parker GA (2009). Sperm competition and sperm phenotype. Sperm Biology.

[58] Birkhead T, Pizzari T (2002). Postcopulatory sexual selection. Nat. Rev. Genet.

[59] Firman RC, Gasparini C, Manier MK, Pizzari T (2017). Postmating female control: 20 years of cryptic female choice. Trends Ecol. Evol..

[60] Wagner RH, Helfenstein F, Danchin E (2004). Female choice of young sperm in a genetically monogamous bird. Proc. R. Soc. Lond. Ser. B.

[61] Trivers RL (2017). Sexual Selection and the Descent of Man.

[62] Parker GA (1979). Sexual selection and sexual conflict. Sex. Sel. Reprod. Competition Insects.

[63] Keller LF, Waller DM (2002). Inbreeding effects in wild populations. Trends Ecol. Evol..

[64] Brouwer L (2010). MHC-dependent survival in a wild population: Evidence for hidden genetic benefits gained through extra-pair fertilizations. Mol. Ecol..

[65] Kempenaers B (2007). Mate choice and genetic quality: A review of the heterozygosity theory. Adv. Stud. Behav..

[66] Neff BD, Pitcher TE (2005). Genetic quality and sexual selection: An integrated framework for good genes and compatible genes. Mol. Ecol..

[67] Bakst M (2011). Physiology and endocrinology symposium: Role of the oviduct in maintaining sustained fertility in hens. J. Anim. Sci..

[68] Stockley P, Gage M, Parker G, Møller A (1997). Sperm competition in fishes: The evolution of testis size and ejaculate characteristics. Am. Nat..

[69] Immler S (2007). The evolution of sperm morphometry in pheasants. J. Evol. Biol..

[70] Kleven O, Laskemoen T, Lifjeld JT (2009). Sperm length in sand martins Riparia riparia: A comment on Helfenstein et al. J. Avian Biol..

[71] Helfenstein F, Szép T, Nagy Z, Kempenaers B, Wagner RH (2008). Between-male variation in sperm size, velocity and longevity in sand martins Riparia riparia. J. Avian Biol..

[72] Du Sert NP (2020). Reporting animal research: Explanation and elaboration for the ARRIVE guidelines 20. PLoS Biol..

[73] Burrows W.H., Quinn J.P. (1937). The Collection of Spermatozoa from the Domestic Fowl and Turkey. Poult. Sci..

[74] Santiago-Moreno J., Castaño C., Toledano-Díaz A., Coloma M.A., López-Sebastián A., Prieto M.T., Campo J.L. (2011). Semen cryopreservation for the creation of a Spanish poultry breeds cryobank: Optimization of freezing rate and equilibration time. Poult. Sci..

[75] Elsayed M, El-Sherry TM, Abdelgawad M (2015). Development of computer-assisted sperm analysis plugin for analyzing sperm motion in microfluidic environments using Image-J. Theriogenology.

[76] Abd-Elhafeez HH (2021). Endocrine, stemness, proliferative, and proteolytic properties of alarm cells in ruby-red-fin shark (Rainbow Shark), Epalzeorhynchos frenatum (Teleostei: Cyprinidae). Microsc. Microanal..

[77] Morris JK (1965). A formaldehyde glutaraldehyde fixative of high osmolality for use in electron microscopy. J. Cell Biol..

[78] Soliman SA, Kamal BM, Abuo-Elhmad AS, Abd-Elhafeez HH (2020). Morphological and histochemical characterization of the dermal plates of pleco (Hypostomus plecostomus). Microsc. Microanal..

[79] Abd-Elhafeez HH, Hassan AHS, Hussein MT (2021). Melatonin administration provokes the activity of dendritic reticular cells in the seminal vesicle of Soay ram during the non-breeding season. Sci. Rep..

[80] Anwar SM, Abd-Elhafeez HH, Abdel-maksoud FM, Abdalla KE (2021). Morph-anatomic and histochemical study of ileum of goose (Alopochen egyptiacus) with special references to immune cells, mucous and serous goblet cells, telocytes, and dark and light smooth muscle fibers. Microsc. Res. Tech..

[81] Abd-Elhafeez HH, Abou-Elhamd AS, Soliman SA (2020). Morphological and immunohistochemical phenotype of TCs in the intestinal bulb of Grass carp and their potential role in intestinal immunity. Sci. Rep..

[82] Abdel Hafeez H, Zaki R, Abd El-Mageed D (2016). Applying light, histochemical and scanning histological methods for the detection of unauthorized animal and herbal content in street meat sandwich: What is in the sandwich we eat. J. Food Process Technol..

[83] Chen C-F (2009). Performance comparison of dwarf laying hens segregating for the naked neck gene in temperate and subtropical environments. Genet. Sel. Evol..

[84] Der G, Everitt BS (2008). A Handbook of Statistical Analyses Using SAS.

[85] Duncan DB (1955). Multiple range and multiple F tests. Biometrics.

