# Supplementary material for: Sperm tendency to agglutinate in motile bundles in relation to sperm competition and fertility duration in chickens
Source: Sci Rep. 2022 Nov 7;12:18860. doi: 10.1038/s41598-022-22049-8 (PMC9640612; doi:10.1038/s41598-022-22049-8)
Supplement: Supplementary file 10 — Supplementary Legends. [file 41598_2022_22049_MOESM10_ESM.doc]

**Sperm tendency to agglutinate in motile bundles in relation to sperm competition and fertility duration in chickens**

**M. A. M. Sayed^1^*, H. H. Abdelhafeez^2^, O. S. Afifi^1^, M. W. Marzouq^3^, Taymour M El-Sherry^4^**

**^1^Department of Poultry Production, Faculty of Agriculture, Assiut University, Assiut, Egypt.71526**

**^2^Department of cell and tissues, Faculty of Veterinary Medicine, Assiut University, Egypt.**

**^3^Department of Poultry Production, Faculty of Agriculture, New Valley University, Egypt. 72712**

**^4^Department of Theriogenology, Faculty of Veterinary Medicine, Assiut University, Egypt.**

**Corresponding Author*:**

**Mohamed Sayed, Faculty of Agriculture, Assiut University, Assiut, Egypt.**

**Tel: 002 088 2185729**

**Fax: 002 088 2331384**

**E-mail: mohamed.sayed4@agr.au.edu.eg**

**Video Legends (supplementary file)**

**Video Legends**

**Video 1: This video shows dozens of Sharkasi sperm assembled into a bundle that increases in**

**size by joining more single sperm and connecting to another bundle of sperm.**

**Video 2: The video shows a group of long, motile sperm bundles of Sharkasi chickens**

**swimming parallel to each other through a flowing fluid in a microfluidic slide.**

**Video 3: The video shows a number of Dandarawi sperm forming thin sperm bundle. The**

**difference in bundle thickness between Dandarawi and Sharkasi is obvious.**

**Video 4: The video shows a number of motile sperm bundles of Dandarawi chickens swimming**

**through a flowing fluid in a microfluidic slide. Note the short length of Dandarawi sperm**

**bundles.**

**Video 5: This video shows Sharkasi sperm bundles when semen was diluted 1 : 40 (v : v) with Lake and Ravie diluent. Note the elaborated network of thick sperm bundles.**

**Video 6: This video shows Sharkasi sperm bundles when semen was diluted 1 : 60 (v : v) with Lake and Ravie diluent. Note the disintegration of the complex network into parallel sperm bundles.**

**Video 7: This video shows the effect of increasing the dilution rate to 1 : 200 (v : v) on Sharkasi sperm bundles. Note dispersal of most bundles and the low motility of lonesome and agglutinated sperm.**

**Video 8: This video shows Dandarawi sperm bundles when semen was diluted 1 : 40 (v : v) with Lake and Ravie diluent. Note the thinner and less cohesive Dandarawi sperm bundles with a complete absence of bundle branching.**

**Video 9: Increasing the dilution rate to 1 : 80 (v : v) caused Dandarawi sperm to die.**
